# Treatment of malignant primary cardiac lymphoma with tumor resection using minimally invasive cardiac surgery

**DOI:** 10.1186/s13019-018-0778-6

**Published:** 2018-09-26

**Authors:** Yuki Endo, Yoshitsugu Nakamura, Miho Kuroda, Yusuke Nakanishi, Yujiro Ito, Takaki Hori, Rumiko Okamoto, Hiroshi Konishi

**Affiliations:** 1 Department of Cardiovascular Surgery, Chiba-Nishi General Hospital, 107-1, Kanegasaku, Matsudo-shi, Chiba, 270-2251 Japan; 2 Departments of Oncology, Chiba-Nishi General Hospital, 107-1, Kanegasaku, Matsudo-shi, Chiba, 270-2251 Japan; 3 Departments of Hematology, Chiba-Nishi General Hospital, 107-1, Kanegasaku, Matsudo-shi, Chiba, 270-2251 Japan

**Keywords:** Malignant primary cardiac lymphoma, Minimally invasive cardiac surgery, Tumor resection, Diffuse large B-cell malignant lymphoma, R-CHOP therapy

## Abstract

**Background:**

Primary cardiac lymphoma (PCL) is extremely rare and progresses rapidly. The treatment of PCL has not yet been established. Unlike lymphoma that arises from other organs, PCL causes cardiovascular events. We report the complete remission (CR) of PCL after tumor resection using minimally invasive cardiac surgery (MICS) and chemotherapy.

**Case presentation:**

The patient was a 79-year-old man who visited our hospital with chief complaints of weight loss and leg edema. A 40 × 30 mm mobile pedunculated tumor continuous with the right ventricular heart muscle was present in the right atrium upon echocardiography and extended cardiac surgery was difficult to perform. Tumor embolism-induced sudden death was prevented and a pathological diagnosis was obtained by making a 4-cm skin incision, and tumor resection with MICS was performed through a right fourth intercostal thoracotomy with a cardiopulmonary system. The histopathological diagnosis was diffuse large B cell malignant lymphoma. Eight cycles of postoperative rituximab plus cyclophosphamide, doxorubicin, vincristine, and prednisone (R-CHOP) therapy were performed. Three years after surgery, the tumor was not visible on imaging and CR was maintained.

**Conclusions:**

This case highlights that tumor resection using MICS is effective for avoiding the risk of sudden death. This technique was useful for the diagnosis and treatment of a malignant cardiac tumor in an elderly patient that required a difficult extended cardiac surgery.

**Electronic supplementary material:**

The online version of this article (10.1186/s13019-018-0778-6) contains supplementary material, which is available to authorized users.

## Background

Benign tumors such as myxomas account for a high percentage of primary cardiac tumors; however, primary malignant cardiac tumors (PMCTs) account for 5.1–28.7% of primary cardiac tumors. Primary cardiac lymphoma (PCL) is extremely rare, accounting for only 1.0–1.6% of PMCTs, but this tumor is difficult to diagnose and progresses rapidly [1, 2]. Moreover, the prognosis is poor because no consistent treatment has been established [1–3]. Unlike lymphoma that arises from other organs, PCL causes cardiovascular events. If a malignant cardiac tumor is suspected, biopsy is necessary to obtain a definitive diagnosis for chemotherapy and radiotherapy and can be achieved with either computed tomography (CT)-guided puncture or an intravenous approach. However, these methods are not able to prevent sudden death due to tumor embolism, can yield inaccurate samples due to tumor necrosis and fibrin clots, and cannot be used for mobile tumors because of the risk of embolus formation. Furthermore, cardiac surgery via a median sternotomy is very invasive, particularly in elderly patients. In addition, radiotherapy cannot be used with median sternotomy due to the risks of postoperative surgical site infections and mediastinitis. Therefore, mobile tumor resection by MICS without a median sternotomy was performed to reduce the risk of tumor embolism and to obtain an accurate pathological diagnosis. We report the CR of PCL after tumor resection using MICS and chemotherapy.

## Case report

The patient was a 79-year-old man with chief complaints of exertional dyspnea, leg edema, and weight loss. On transthoracic echocardiography (TTE), a 25 × 40 mm mobile pedunculated mass continuous with the right ventricular heart muscle was detected in the right atrium and the patient was admitted to our department for close examination and treatment. At admission, his height was 162.0 cm, body weight was 61.1 kg, body temperature was 36.3 °C, pulse was 62 beats/min, blood pressure was 112/59 mmHg, and SpO_2_ was 100% (room air). Pulmonary sounds were clear with no crackles, and heart sounds were regular with no murmur. Leg edema was present.

Plain chest radiography revealed a cardiothoracic ratio of 49% with no cardiac dilation. Electrocardiography revealed a sinus rhythm with a heart rate of 71 beats/min with nonspecific ST-T segment changes. Blood chemistry revealed the following: white blood cell (WBC) count of 51.9 × 10^4^/μL, hemoglobin (Hb) of 14.9 g/dL, platelet (Plt) count of 16.3 × 10^4^/μL, creatine kinase (CK) of 81 U/L, creatine kinase-MB (CKMB) of 8 ng/mL, lactate dehydrogenase (LDH) of 161 U/L, C-reactive protein (CRP) of 0.10 mg/dL, carcinoembryonic antigen (CEA) of 0.7 ng/mL, prostate-specific antigen (PSA) of 1.2 ng/mL, squamous cell carcinoma (SCC) antigen of 1.2 ng/mL, and soluble IL-2 receptor: 633 U/mL. Inflammatory parameters were within the normal range and the soluble IL-2 receptor level was slightly elevated, but the levels of other tumor markers were within their normal ranges. A coronary computed tomography (CT) scan showed no significant stenosis. It was deemed very difficult to completely excise, so we decided on partial tumor resection with MICS to reduce the risk of tumor embolism and to obtain an accurate pathology diagnosis. Therefore, we did not perform CAG. If we had performed a CAG, we may have seen arteries feeding the tumor.

**Fig. 1:**
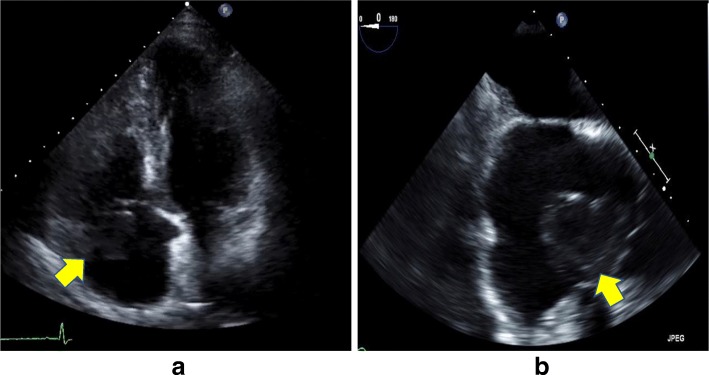
Preoperative echocardiography. **a** Transthoracic echocardiography showed a mobile pedunculated tumor in the right atrium. **b** Transesophageal echocardiography showed a solid, septated tumor with an irregular surface invading the free wall of the right atrium and surrounding the annulus of the anterior cusp and right and left coronary cusps of the aortic valve

**Fig. 2:**
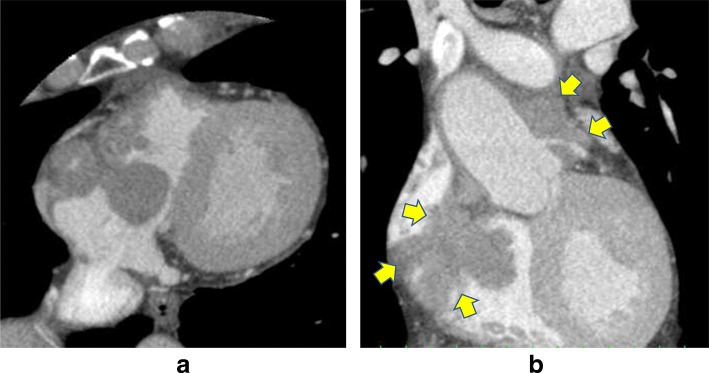
Preoperative contrast-enhanced CT. The big tumor was existed in the right atrium with axial section (**a**). The right arrows show invasion based on soft tissue intensity near the tricuspid valve above the anterior right ventricle in the region between the aorta and pulmonary artery, and around the pulmonary artery with coronal section (**b**)

**Fig. 3:**
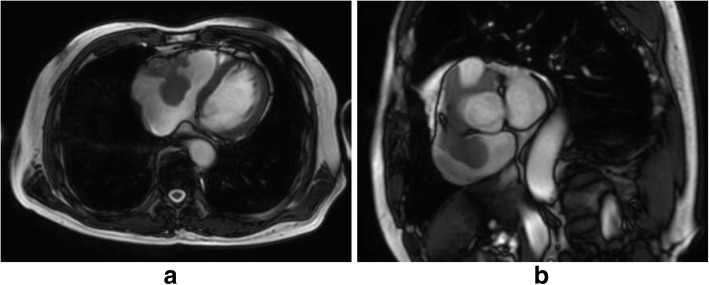
Preoperative cardiac MRI. Cardiac MRI showed a thickened anterior wall near the tricuspid valve with axial section (**a**) and a mass protruding into the lumen and expanding into the region between the aorta and pulmonary artery with coronal section (**b**)

**Fig. 4:**
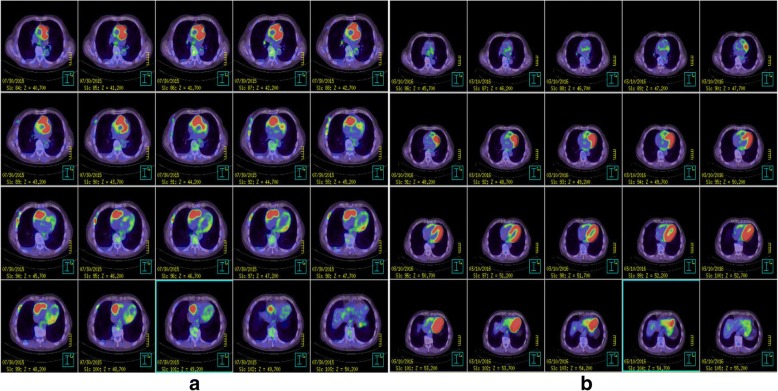
F-18 FDG-PET. **a** Preoperative PET-CT showed abnormal accumulation in the right atrium surroundings the aortic root. **b** Postoperative PET-CT showed complete remission after completion of chemotherapy. Accumulation in the left ventricular myocardium was physiological, and the abnormal accumulation around the aortic root and pulmonary artery had disappeared

TTE showed a 40 × 30-mm mobile pedunculated tumor in the right atrium that was continuous with the right ventricular heart muscle (Fig. 1a). Transesophageal echocardiography (TEE) showed a solid septated tumor with an irregular surface invading the free wall of the right atrium and surrounding the annulus of the anterior cusp and right and left coronary cusps of the aortic valve (Fig. 1b). Contrast-enhanced CT showed invasion based on soft tissue intensity near the tricuspid valve above the anterior right ventricle in the region between the aorta and pulmonary artery and around the pulmonary artery (Fig. 2a, b). Cardiac magnetic resonance imaging (MRI) showed a thickened anterior wall near the tricuspid valve and a mass protruding into the lumen and expanding into the region between the aorta and the pulmonary artery (Fig. 3a, b); in addition to the patient’s advanced age, these features made it difficult to perform extended cardiac surgery. On fluorodeoxyglucose positron emission tomography (F-18 FDG-PET), there was abnormal accumulation in the right atrium surrounding the aortic root (Fig. 4a).

**Fig. 5:**
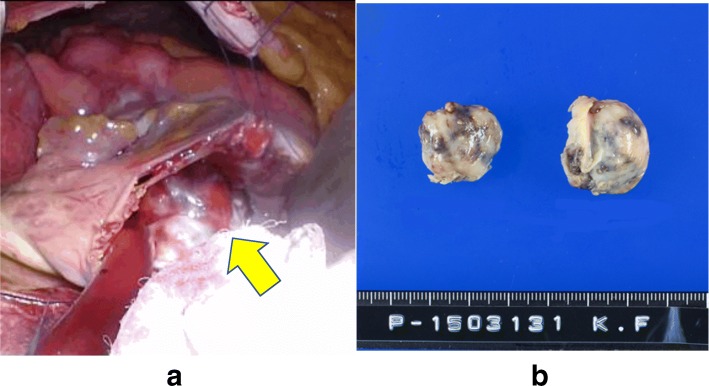
Intraoperative photos and specimen. **a** An oblique incision was made in the right atrium with the heart beating, and the lumen was observed. The tumor adhered to the anterior surface of the right atrium but not to the annular region and had marked mobility. **b** Intraoperative macroscopic findings revealed a tumor with a smooth, greyish-white surface

Surgery was performed to prevent tumor embolism-induced sudden death and to obtain a pathological diagnosis. Anticoagulation was not performed preoperatively because it was unlikely that the tumor was a thrombus. A double-lumen tube was inserted during surgery and a Swan-Ganz catheter and 14-Fr cannula were inserted through the right internal jugular vein after draping. In a supine position with 30° elevation of the right side, a 4-cm skin incision was made in the fourth intercostal region at the medial aspect of the nipple. Meanwhile, the femoral artery (FA) and femoral vein (FV) were exposed. A pericardiotomy was performed 2 cm anterior to the phrenic nerve and the pericardium was elevated. After systemic heparinization, an 18-Fr blood supply tube was inserted through the right FA, and a 25-Fr cannula was inserted through the right FV to establish a cardiopulmonary bypass (CPB). The superior vena cava was blocked with a bulldog clamp and the heart rate was controlled at 40–50 bpm with a β-blocker. An oblique incision was made in the right atrium with the heart beating, and the lumen was observed. The tumor adhered to the anterior surface of the right atrium but not to the annular region and had marked mobility. The tumor was grasped with an Endocatch and the pedicle of 1-cm width was transected using electric cautery (Fig. 5a). The lack of any residual right atrial tumor or shunt was confirmed and the right atrium was closed in a double suture pattern; the patient was then weaned from CPB. The pericardium was closed as far as possible, the wound was closed by the standard method, and surgery was completed. The operative time was 1 h 56 min, and the duration of CPB was 38 min (Additional file 1).

**Fig. 6:**
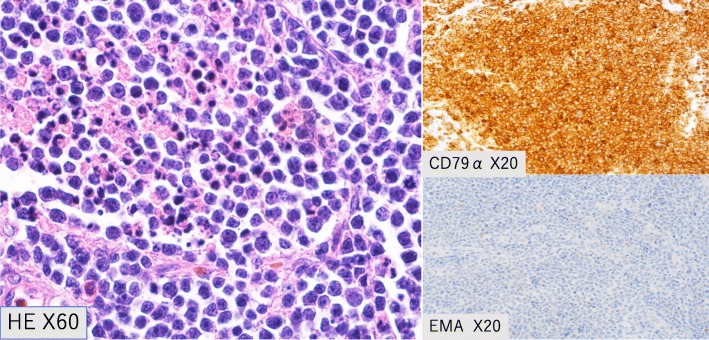
Pathology. The tumor cells were CD79α-positive and CD3-negative. B-cell-derived cells were overwhelmingly predominant, suggesting that the lesion was a B-cell-derived tumor. Epithelial membrane antigen immunostaining was negative

Intraoperative macroscopic findings revealed a tumor with a smooth, greyish-white surface (Fig. 5b). A blackish-brown region suggestive of hemorrhage was present inside and on pathological examination (Fig. 6), a diffuse proliferation of round cells with a high nuclear-to-cytoplasmic (N/C) ratio were observed on hematoxylin and eosin staining. The tumor cells were mainly medium- and small-sized cells that contained nuclei with a shallow cut that were the same size or slightly smaller than the nuclei of vascular endothelial cells; large cells were also present. Broken nuclear products and histiocytes phagocytosing these products were also observed. Upon immunohistological staining, the tumor cells were CD79α-positive and CD3-negative. B-cell-derived cells were overwhelmingly predominant, which suggested that the lesion was a B-cell-derived tumor. Epithelial membrane antigen (EMA) immunostaining was negative. Based on these findings, the patient was diagnosed with diffuse large B-cell lymphoma (DLBCL).

Extubation was performed 6 h after surgery and the patient was transferred to a general ward 2 days after surgery. The disappearance of the tumor from the annular region was confirmed on TTE 5 days after surgery and the patient was discharged when he was able to independently walk 6 days after surgery. After observation at an outpatient clinic, rituximab plus cyclophosphamide, doxorubicin, vincristine, and prednisone (R-CHOP) therapy was initiated 37 days after surgery. After 8 cycles were administered in total, an FDG-PET scan performed 456 days after surgery showed no abnormal accumulation (Fig. 4b), indicating CR.

## Discussion

PMCTs account for approximately 5.1–28.7% of primary cardiac tumors and diagnosis is difficult in many cases [1, 2]. PMCTs tend to occur in young patients with a mean age of 44 years and there was no difference in the ratio between the sexes [3]. The prognosis is poor, with a five-year survival rate of 19% [4]. Among PMCTs, although 75% is sarcoma, 1.0–1.6% is PCL. PCL is defined as extranodal lymphoma in which lesions only develop in the heart and pericardium or in which a giant tumor develops in the heart accompanied by an asymptomatic solitary extracardiac lesion or several localized lesions [5, 6]. Common sites are the right heart and epicardium, and the histological type is DLBCL in many cases [6]. Unlike lymphoma arising from other organs, PCL causes cardiovascular events such as heart failure, cardiac tamponade, arrhythmia, and embolism, leading to an acute presentation [1–7].

In our patient, a giant mobile pedunculated tumor in the right atrium with heart muscle invasion was evident on TTE and TEE performed to evaluate the causes of the patient’s difficulty breathing and leg edema. An intracardiac tumor and invasion of the region between the aorta and pulmonary artery and near the pulmonary artery were observed without abnormalities in other organs on CT, which suggested a PMCT. In addition, PCL was suspected because of heart failure symptoms such as difficulty breathing and leg edema, and the development in the right heart.

There were three therapeutic options: 1. complete resection through median sternotomy, 2. CT-guided or intravenous biopsy followed by chemotherapy or radiotherapy, and 3. tumor resection of the mobile part with MICS followed by chemotherapy or radiotherapy. Regarding complete resection through median sternotomy, this was deemed too invasive for the patient because the cardiac invasion pattern of the tumor was complex, would require resection of the atrioventricular junction, and the patient was elderly. In addition, median sternotomy is associated with a high risk of surgical site infections and mediastinitis if radiotherapy on the mediastinum was necessary. Certainly, there was a case report in which the tumor was resected with an emergency median sternotomy; however, the results of enlargement surgery via a median sternotomy incision are poor and there has never been a good report [8, 9]. Regarding CT-guided or intravenous biopsy, this could not prevent sudden death due to tumor embolism and atrioventricular valve obstruction. Generally, biopsy could have been achieved with a CT-guided or intravenous approach and some reports have shown these approaches’ effectiveness [8–10]. However, these techniques were not possible in this case because the tumor was very mobile; in addition, these methods can yield inaccurate samples due to tumor necrosis and fibrin clots [10].

Based on these considerations, to avoid the risk of embolism and perform a biopsy in a minimally invasive manner, we decided to perform tumor resection using minimally invasive beating-heart surgery followed by adjuvant therapy. Preoperative FDG-PET showed abnormal accumulation in the right atrium surrounding the aortic root and the pathological diagnosis was DLBCL. This disease responds to anthracycline-based chemotherapy regimens [11] and remission has been reported after chemotherapy alone or after chemotherapy used in combination with radiotherapy [12, 13] The DLBCL treatment algorithm for our patient recommended combined modality treatment with 3 cycles of R-CHOP therapy with involved-field radiotherapy (IFRT) or 6–8 cycles of R-CHOP therapy ± IFRT [14–16]. Early diagnosis and treatment intervention may determine the outcome [13], and 8 cycles of R-CHOP therapy were performed to avoid radiotherapy, beginning 37 days after MICS tumor resection. There was no abnormal accumulation on FDG-PET after completing chemotherapy at 456 days after surgery and CR was achieved. IFRT was not performed because the radiation range was unclear and damage to the heart muscle by direct irradiation was possible. Careful observation has been recommended for patients with CR after R-CHOP therapy because there are no data showing improved survival with maintenance therapy [17]. Our patient has had no recurrence and careful follow-up is ongoing.

## Conclusion

This case provides an example of a patient with an accurate pathological diagnosis who underwent successful chemotherapy and in whom sudden death by tumor embolism was avoided by tumor resection with MICS rather than by median sternotomy, which is associated with risks of delayed wound healing and mediastinitis if postoperative radiotherapy is required. As in this patient, tumor resection with MICS may be useful for patients in whom sudden death by tumor embolism is highly likely and in whom chemotherapy, radiotherapy, or both are necessary.

## Additional file

Additional file 1: **Movie S1.** Operation movie. (MOV 139246 kb)
